# Converging Evidence for the Processing Costs Associated with Ambiguous Quantifier Comprehension

**DOI:** 10.3389/fpsyg.2013.00153

**Published:** 2013-04-02

**Authors:** Corey T. McMillan, Danielle Coleman, Robin Clark, Tsao-Wei Liang, Rachel G. Gross, Murray Grossman

**Affiliations:** ^1^Frontotemporal Degeneration Center, Department of Neurology, Perelman School of Medicine, University of PennsylvaniaPhiladelphia, PA, USA; ^2^Department of Linguistics, University of PennsylvaniaPhiladelphia, PA, USA; ^3^Department of Neurology, Thomas Jefferson UniversityPhiladelphia, PA, USA

**Keywords:** language, quantifiers, fMRI, volumetric MRI, frontotemporal dementia

## Abstract

Traditional neuroanatomic models of language comprehension have emphasized a core language network situated in peri-Sylvian cortex. More recent evidence appears to extend the neuroanatomic network beyond peri-Sylvian cortex to encompass other aspects of sentence processing. In this study, we evaluate the neuroanatomic basis for processing the ambiguity in doubly-quantified sentences. For example, a sentence like “All the dogs jumped in a lake” can be interpreted with a collective interpretation (e.g., several dogs jumping into a single lake) or a distributive interpretation (e.g., several dogs each jumping into a different lake). In Experiment 1, we used BOLD fMRI to investigate neuroanatomic recruitment by young adults during the interpretation of ambiguous doubly-quantified sentences in a sentence-picture verification task. We observed that young adults exhibited a processing cost associated with interpreting ambiguous sentences and this was related to frontal and parietal cortex recruitment. In Experiment 2, we investigate ambiguous sentence processing with the identical materials in non-aphasic patients with behavioral variant frontotemporal dementia (bvFTD) who have frontal cortex disease and executive and decision-making limitations. bvFTD patients are insensitive to ambiguity associated with doubly-quantified sentences, and this is related to the magnitude of their frontal cortex disease. These studies provide converging evidence that cortical regions that extend beyond peri-Sylvian cortex help support the processing costs associated with the interpretation of ambiguous doubly-quantified sentences.

## Introduction

Neuroanatomic models of language comprehension traditionally implicate a core peri-Sylvian language network in sentence processing. Much evidence has been accumulated to suggest that “Broca’s area” located in left inferior frontal cortex (IFC)and left posterolateral temporal cortex (plTC) – so-called Wernicke’s area – support the grammatical and semantic relationships between sentence constituents (Friederici and Gorrell, 1998; Friederici et al., 2003; Grodzinsky and Friederici, 2006; Grodzinsky and Santi, 2008). However, many sentences depend on additional processing resources, such as sentences that are ambiguous. In this paper we provide converging evidence for the neural basis for these additional resources from an fMRI study of healthy adults and a behavioral study with non-aphasic patients who have a focal neurodegenerative disease that compromises these resources.

In the present study, we focus on the class of ambiguous sentences involving quantifiers. A quantifier comes from a class of words that expresses a referent’s quantity. There are at least three sub-classes of quantifiers (McMillan et al., 2005, 2006; Troiani et al., 2009a,b). First-order quantifiers like “at least three” appear to depend directly on number knowledge. We have empirically demonstrated that focal neurodegenerative patients with parietal disease due to corticobasal syndrome (CBS) and posterior cortical atrophy (PCA) have highly correlated deficits understanding first-order quantifiers and precise numbers (McMillan et al., 2006; Troiani et al., 2009a; Morgan et al., 2011), and regression analyses relate this impairment directly to parietal disease. Likewise, comprehension of first-order quantifiers is associated with activation of parietal cortex in fMRI studies of healthy adults (McMillan et al., 2005; Troiani et al., 2009a; Heim et al., 2012). A second sub-class of quantifiers like “less than half” are higher-order. These depend on number knowledge plus strategic executive resources and working memory. Higher-order quantifiers are additionally associated with dorsolateral prefrontal cortex (dlPFC), as shown by fMRI activation in healthy adults (McMillan et al., 2005) and deficits following neurodegenerative disease in prefrontal regions (McMillan et al., 2006; Troiani et al., 2009a; Morgan et al., 2011). A third class of quantifiers like “all” and “some” are Aristotelean, and depend on a simple attentional mechanism associated with medial PFC regions (Troiani et al., 2009a).

While prior work has been limited to investigating unambiguous quantifiers, a quantifier is often ambiguous when co-occurring in the same sentence as a second quantifier, that is, when the sentence is “doubly-quantified.” Consider the following examples:

(1A) All the dogs jumped in a lake.
(1B) All the dogs jumped in a puddle.

Sentences 1A and 1B contain two quantifiers, the logical quantifier “all” and the existential quantifier “a,” and as a result are ambiguous. Both of these sentences can be interpreted with either a collective interpretation (e.g., several dogs jumping into a shared lake or puddle) or a distributive interpretation (e.g., each of several dogs jumping into its own lake or puddle). While it has been suggested that individuals prefer a collective interpretation for doubly-quantified sentences containing “all” (Ioup, 1975), both interpretations are plausible and therefore one can hypothesize that readers incur a processing cost associated with interpreting these sentences.

Psycholinguistic researchers have debated whether ambiguous sentences are processed in a serial manner where a preferred interpretation is developed during the course of processing (Ferreira and Clifton, 1986; Garnsey et al., 1997), or a parallel manner where the ultimate interpretation is achieved at the end of the sentence (MacDonald, 1994; MacDonald et al., 1994; Gibson and Pearlmutter, 1998; Spivey and Tanenhaus, 1998). Regardless of the preference for a serial or parallel approach to sentence processing, doubly-quantified sentences that are more ambiguous appear to be associated with an increased processing cost. Empirical evidence supporting the increased cost associated with more ambiguous doubly-quantified sentences comes from several sources. An ERP study observed that less-preferred interpretations of doubly-quantified sentences do not yield typical semantic responses such as a N400, but instead are associated with a slow wave negative shift (Dwivedi et al., 2010). An eye-tracking study found that participants show delayed reading times when they encounter a less-preferred resolution of a doubly-quantified sentence compared to a more preferred interpretation (Filik et al., 2004).

Investigations of the neuroanatomic basis for the processing cost associated with the comprehension of more ambiguous sentences may be clarified by evaluating the interpretation of doubly-quantified sentences. Some fMRI studies have shown increased activation of the core sentence processing system during comprehension of ambiguous sentences. For example, higher levels of brain activation were observed in left IFC and left plTC when an ambiguous sentence was resolved in favor of either the more preferred or less-preferred interpretation, as compared to an unambiguous sentence (Mason et al., 2003). The authors explained their findings on the basis of a ranked parallel model that increases activation throughout the core sentence processing network to maintain multiple possible sentence interpretations.

Neuroimaging studies of healthy adults and voxel-based morphometric assessments of patients with focal neurodegenerative diseases have suggested an alternate approach to the neuroanatomic basis of sentence processing. This implicates brain regions beyond those involved in the traditional peri-Sylvian model of language processing. From this perspective, the core peri-Sylvian language network in the left hemisphere may recruit additional resources as needed to support sentence processing (Wingfield and Grossman, 2006). For example, some sentences in real-world conversations can be quite lengthy, and additional working memory resources may be necessary in order to support the processing demands associated with these sentences. In fMRI studies of healthy adults, we and others have found that lengthy sentences recruit dorsal portions of IFC outside of the traditional peri-Sylvian language network to support working memory (Caplan and Waters, 1999; Cooke et al., 2002, 2006; Kaan and Swaab, 2002; Fiebach et al., 2005). This area is similar to that activated in fMRI studies of verbal working memory (Smith and Jonides, 1999; Smith et al., 2001). Moreover, converging evidence comes from non-aphasic patients with working memory limitations who have difficulty interpreting lengthy sentences, and this impairment is associated with disease in dorsal portions of IFC important for working memory (Cooke et al., 2003; Peelle et al., 2008).

The interpretation of ambiguous sentences also may implicate brain regions that are outside of core peri-Sylvian sentence processing areas. Previous fMRI work in healthy adults in our lab thus associated the disambiguation of sentences containing a temporary structural ambiguity with dlPFC (Novais-Santos et al., 2007). We related this to an on-line decision-making mechanism that estimates the more likely interpretation of a sentence’s ambiguous structure based on the probabilities associated with the biases of the sentence’s verb. We also observed upregulation of dlPFC when evaluating the probabilistic likelihood of an ambiguous pronoun’s referent (McMillan et al., 2011). More recently, an fMRI study reported activation of decision-making mechanisms in PFC during the resolution of doubly-quantified sentences (McMillan et al., 2011). However, this fMRI evidence required participants to make a forced choice of either a distributive or collective interpretation, and our finding may simply have reflected activations needed for task performance.

In the present study, we investigated the neuroanatomic basis for the resolution of doubly-quantified sentence meaning in two experiments. First, we performed a BOLD fMRI study with healthy young adults who were asked to verify whether a doubly-quantified sentence accurately describes a scene representing either a collective or a distributive interpretation. We manipulated the transparency with which the sentence reflected the collective or distributive character of the picture. Second, we used identical stimulus materials to investigate the comprehension of doubly-quantified sentences in behavioral variant frontotemporal dementia (bvFTD) patients, and we related their performance to the anatomic distribution of their gray matter atrophy. These patients do not have aphasia and have relatively preserved grammar and semantics. However, they have executive and social decision-making limitations associated with prefrontal cortex disease (Libon et al., 2007; Rascovsky et al., 2011; McMillan et al., 2012b). We hypothesized that comprehension of ambiguous, doubly-quantified sentences will incur additional processing costs, reflected by increased response times, that rely on both language resources in peri-Sylvian cortex and additional fronto-parietal resources that extend beyond a core language network.

## Experiment 1: BOLD fMRI Study in Healthy Adults

### Methods

#### Participants

Seventeen young adults were recruited from the University of Pennsylvania community and financially compensated for their participation. All participants were right-handed, native-English speakers with a negative history of neurological or psychiatric disorders. See Table 1 for a summary of demographic characteristics. Informed consent was provided by all participants according to a protocol approved by the Institutional Review Board at the University of Pennsylvania. One participant was excluded from all analyses for responding with the same button-press for every experimental trial and therefore all analyses include 16 participants.

**Table 1:** **Mean (Standard Error) demographic characteristics of (A) healthy young adults in the functional MRI experiment (Experiment 1) and (B) healthy seniors and behavioral variant frontotemporal dementia (bvFTD) patients from behavioral Experiment 2**.

Experiment	*N*	Age	Education	MMSE
**(A) EXPERIMENT 1: BOLD fMRI**				
Healthy young adults	16	23.4 (0.6)	16.3 (0.4)	–
**(B) EXPERIMENT 2: BEHAVIORAL PATIENT STUDY**				
bvFTD	16	64.3 (2.0)	15.4 (0.8)	24.7 (1.2)
Healthy Seniors	16	64.4 (2.0)	14.3 (0.5)	29.1 (0.3)

#### Experimental stimulus materials

We generated a total of 54 ambiguous doubly-quantified sentences such as “All the dogs jumped in a puddle/lake.” In one-third of the sentences the final noun was a smaller object (e.g., “puddle”) consistent with a distributive interpretation and one-third of the sentences contained a larger object (e.g., “lake”) to yield a collective interpretation. To minimize the possibility that participants developed a heuristic for responding, we also included a midsize object (e.g., “pond”) in one-third of our stimuli. Since all experimental stimuli were identical with the exception of the final noun (e.g., “All the dogs jumped in a ___.”) we only evaluated the psycholinguistic properties of the final noun across conditions.

We additionally generated two sets of baseline materials. First, we generated 54 unambiguous baseline sentences that explicitly disambiguated between a collective and a distributive interpretation. Among the unambiguous baseline sentences were sentences that used “same” (e.g., “All the dogs jumped in the same lake”), “different” (e.g., “All the dogs jumped in a different lake”), and “the” (e.g., “The dogs jumped in a lake”). Second, we developed 54 filler sentences that only differed from the stimuli described above by containing the quantifier “every” substituted for “all.” These filler materials containing “every” were intended to obscure to participants that quantified sentences are ambiguous and thus the focus of the experiment. For all analyses we average across all three unambiguous baseline subtypes.

#### Experimental procedure

**Figure 1:**
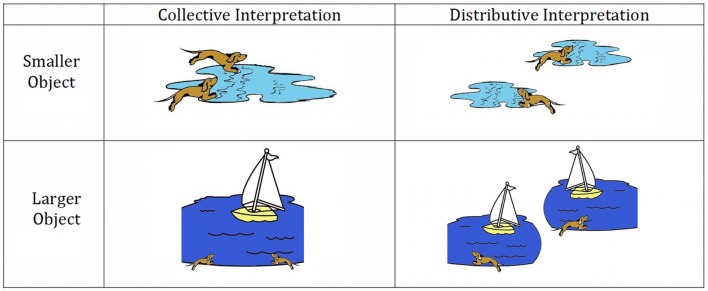
**Sample stimulus images for experimental sentence materials**.

Each experimental trial was comprised of three events, each lasting the duration of 1 TR (3000 ms): (1) an inter-stimulus interval consisting of a blank white screen (2500 ms) and a fixation cross (500 ms) to orient attention; (2) a stimulus sentence presentation (3000 ms); and (3) representation of the stimulus sentence along with a picture illustrating either a collective or a distributive interpretation of the sentence (see Figure 1). Participants were instructed to decide whether the sentence described the picture. Participants were given up to 3000 ms for their judgments.

Prior to the MRI scanning session, participants were given a practice session containing 10 trials to familiarize them with the form of the stimulus materials and the task, and we allowed them to ask questions before entering the scanner. These stimulus items were not re-presented in the experimental task.

All 162 stimuli (54 ambiguous doubly-quantified sentences; 54 unambiguous baselines sentences; 54 “every” filler sentences) were pseudo-randomly distributed across three blocks of equal duration (8.1 min) so that there were equal numbers of each condition within each block. Within each block, we additionally included 11 (15%) randomly distributed null events consisting of a blank screen for the duration of 1 TR (3000 ms). Participants were provided with a 2-min break between each block.

Sentences were presented in black font on a white background using a mirror projection system connected to the computer running E-Prime presentation software. Using a fiber optic response pad (FORP), we monitored whether participants endorsed a sentence-picture pair and we monitored their response latency. The FORP rested on the participant’s lap and contained four buttons oriented in a left-right fashion. To minimize the confound associated with lateralized button-pressing for a particular category of response, half of the participants pushed the leftmost button with their left forefinger for an endorsement and the rightmost button with their right forefinger for a rejection. The FORP was reversed for the other half of the participants.

We monitored participants’ response times to ambiguous doubly-quantified sentences and unambiguous baseline sentences. We eliminated large outlier responses that were greater than 2.5 standard deviations from each individual participants’ overall mean response time, resulting in a removal of 1.7% of total responses. Since both a “yes” and “no” response can be considered “correct” for ambiguous items we included all response times in our analyses. For all analyses we evaluated log-transformed reaction times. A separate analysis restricted to “yes”-only responses revealed a similar statistical result.

#### Neuroimaging acquisition and analysis

Scans were acquired on a Siemens 3.0 T Trio scanner. Each session began with acquisition of a high-resolution T1-weighted structural volume using an MPRAGE protocol (TR = 3000 ms, TE = 3 ms, flip angle = 15°, 1 mm slice thickness, 192 × 256 matrix, resolution = 0.9766 mm × 0.9766 mm × 1 mm). We collected whole-brain BOLD volumes containing 42 axial slices and acquired with fat saturation, 3 mm isotropic voxels, flip angle of 15°, TR = 3 s, TEeff = 30 ms, and a 64 × 64 matrix.

BOLD fMRI data preprocessing and statistical analyses were performed using SPM8 (Wellcome Trust Centre for Functional Neuroimaging, London, UK). We first modeled each individual participant’s data. Low-frequency drift was removed with high-pass filtering with a cutoff period of 128 s and autocorrelations modeled using a first-order autoregressive model. Whole-brain volumes for each participant were realigned to the first volume in the series (Friston et al., 1995) and coregistered with the structural volume (Ashburner and Friston, 1997). After realignment, we inspected each participant’s motion in all directions to verify that no participants had excessive motion artifact, defined as more than 3 mm movement in any axis. The transformation required to bring a participant’s images into standard MNI152 space was calculated using tissue probability maps (Ashburner and Friston, 1997), and these warping parameters were then applied to all functional brain volumes for that participant. During spatial normalization, functional data were interpolated to isotropic 2 mm voxels. The data were spatially smoothed with an 8 mm FWHM isotropic Gaussian kernel.

For each stimulus category, hemodynamic response was estimated by convolving the onset times with a canonical hemodynamic response function. A general linear model approach was used to calculate parameter estimates for each variable for each subject, and linear contrasts for comparisons of interest. These estimates were then entered into second-level random effects analyses to allow us to make inferences across participants.

Our whole-brain fMRI analysis was performed using a one-sample *t*-test in SPM8. We evaluated the activation for ambiguous doubly-quantified sentences relative to unambiguous sentences for the event that included the sentence and picture presentation. This high-level baseline allowed us to subtract out the resources associated with sentence reading and picture processing in an effort to isolate the additional processing demands associated with the comprehension of ambiguous sentences. We report a FDR-corrected height threshold (*q* < 0.05) and included clusters exceeding an extent threshold of 20 adjacent voxels.

### Results

#### Behavioral results

**Figure 2:**
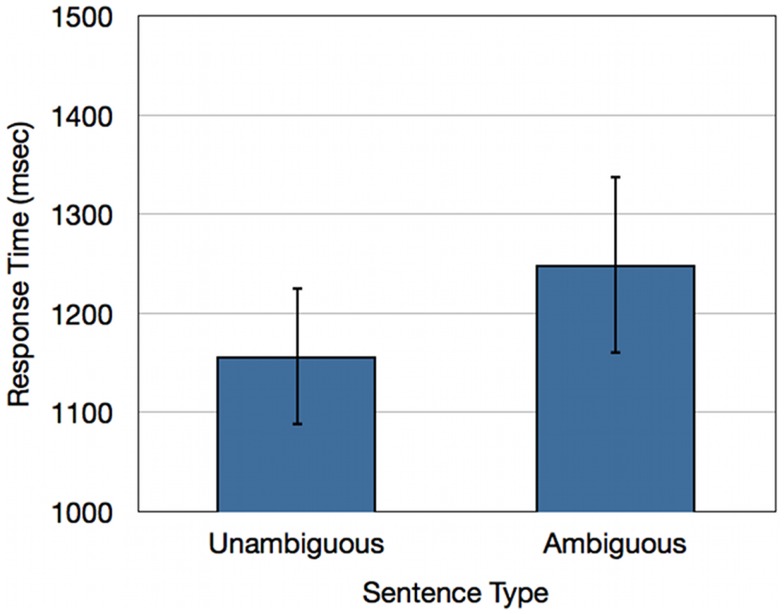
**Response times (ms) for healthy young adults for unambiguous baseline sentences and ambiguous doubly-quantified sentences**.

An analysis of response latencies revealed that participants respond on average 93 ms slower for ambiguous doubly-quantified sentences relative to unambiguous sentences [*t*(17) = 2.74; *p* < 0.05](Refer to Figure 2).

#### fMRI results

**Figure 3:**
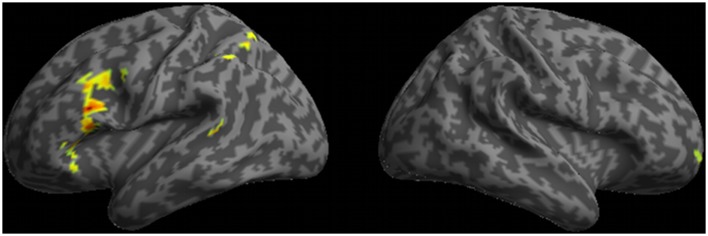
**Regions of activation observed in whole-brain analysis for ambiguous doubly-quantified sentences minus baseline unambiguous sentences**.

A whole-brain analysis of doubly-quantified sentences relative to unambiguous baseline sentences revealed activation of five cortical regions (see Figure 3 and Table 2). This included peri-Sylvian regions in left IFC and left plTC. We additionally observed activation beyond peri-Sylvian cortex that extended from left IFC into left dlPFC and also included other clusters in right rostral PFC (rPFC), left inferior parietal cortex, and dorsomedial PFC.

**Table 2:** **Regions of activation for doubly-quantified sentences minus baseline sentences (*p* < 0.05 FDR-corrected; 20 voxel extent)**.

Neuroanatomic Region (BA)	L/R	Peak MNI coordinate			*Z*-score	Voxels
		*x*	*y*	*z*	
Inferior parietal (40/7)	L	−32	−60	54	4.02	209
Rostral prefrontal (10)	R	24	60	−6	3.74	28
Posterior-lateral temporal (22)	L	−54	−44	8	4.01	40
Inferior frontal (45)	L	−46	18	20	4.88	906
Dorsomedial prefrontal (6)	M	4	16	54	3.94	30

### Experiment 1 interim discussion

Our fMRI results are broadly consistent with neuroimaging studies of language comprehension that have implicated plTC and IFC in a core peri-Sylvian language network (Friederici et al., 2003; Grodzinsky and Friederici, 2006; Grodzinsky and Santi, 2008). As in a previous study (Mason et al., 2003), we observed increased activation of this network for ambiguous sentences compared to unambiguous sentences. Importantly, we found that additional brain regions are recruited as well during subjects’ processing of doubly-quantified sentences, implicating these extra-Sylvian brain regions in support of the additional resources needed to interpret ambiguous sentences.

The extra-Sylvian regions activated for ambiguous sentences compared to unambiguous sentences included right rPFC, left dlPFC, dorsomedial prefrontal cortex, and left parietal cortex. To further evaluate the necessary role of these regions for comprehending ambiguous doubly-quantified sentences we presented identical stimulus materials to non-aphasic patients with bvFTD. These patients typically have disease in frontal and anterior temporal regions (Pereira et al., 2009; Whitwell et al., 2010), and have executive, decision-making, and social limitations despite relatively preserved language abilities (Gleichgerrcht et al., 2010; Rascovsky et al., 2011; McMillan et al., 2012b). A deficit interpreting ambiguous sentences in these non-aphasic patients would lend additional support to the claim that the core peri-Sylvian sentence processing system recruits additional resources to support the processing costs associated with the interpretation of ambiguous sentences.

## Experiment 2: Behavioral-Imaging Correlation Study in Patients with Behavioral Variant Frontotemporal Degeneration

### Methods

#### Participants

We recruited 16 bvFTD patients from the Frontotemporal Degeneration Center at the University of Pennsylvania. All patients were diagnosed by a board-certified neurologist using published consensus criteria (Rascovsky et al., 2011). Other causes of dementia and cognitive difficulty were excluded by clinical exam, blood, and neuroimaging tests. We additionally recruited 16 healthy seniors from the community as a behavioral control group. Healthy seniors and bvFTD patients were demographically comparable for age [*t*(30) < 1; ns] and education [*t*(30) = 1.25; ns]. An evaluation of overall dementia severity was performed using the 30-point scale Mini-Mental Status Examination (MMSE), and this revealed that bvFTD patients had lower MMSE scores than healthy seniors [*t*(30) = 3.61; *p* < 0.001]. Nevertheless, bvFTD patients averaged within the “non-demented” range on this scale (Mean MMSE = 25). Table 1 summarizes clinical and demographic features. Informed consent was provided by all participants according to a protocol approved by the Institutional Review Board at the University of Pennsylvania.

High-resolution volumetric neuroimaging was available for a subset of bvFTD patients (*N* = 11), as described below. MRI images were not available for the remaining bvFTD patients due to health and safety exclusion criteria, including claustrophobia and metallic objects in the body (e.g., pacemakers and shrapnel). We additionally recruited 14 age- [*t*(23) = 1.34; ns] and education- [*t*(23) < 1; ns] matched healthy seniors from the community as a structural neuroimaging control group.

#### Stimulus materials and design

We used identical stimulus materials to those administered in the fMRI experiment above, including 54 doubly-quantified experimental stimuli (equally divided among small and large objects), 54 unambiguous baseline stimuli, and 54 filler sentences containing “every” were used in this experiment. Half of the pictures illustrated a distributive interpretation and half a collective interpretation. Participants were instructed to decide whether the sentence describes the picture. Each sentence stimulus was presented for 3000 ms followed by the presentation of the sentence together with a picture depicting a particular interpretation. Participants were given a maximum of 10 s to respond to each stimulus item. We analyzed log-transformed response times for responding to ambiguous doubly-quantified and unambiguous sentence materials. We eliminated large outlier responses that were greater than 2.5 standard deviations from each individual participants’ overall mean response time, resulting in a removal of 4.0% of total responses. Since both a “yes” and “no” response can be considered “correct” for ambiguous items we included all response times in our analyses. A separate analysis restricted to “yes”-only responses revealed a similar statistical result.

#### Neuroimaging acquisition and analysis

High-resolution T1-weighted three-dimensional spoiled gradient echo images were acquired with repetition time = 1,620 ms, echo time = 3 ms, slice thickness 1.0 mm, flip angle 15°, matrix = 192 × 256, and in-plane resolution 0.9 mm × 0.9 mm. All images were preprocessed using PipeDream^1^ and Advanced Normalization Tools (ANTs)^2^ to perform the most stable and reliable multivariate normalization and structure-specific processing currently available (Avants et al., 2008, 2010; Klein et al., 2010). PipeDream deforms each individual dataset into a standard local template space in a canonical stereotactic coordinate system. Core processing involves mapping T1 structural MRI to a population-specific local template. A diffeomorphic deformation was used for registration that is symmetric so that it is not biased toward the reference space for computing the mappings, and topology-preserving to capture the large deformation necessary to aggregate images in a common space. These algorithms allow template-based priors to guide a calculation of gray matter probability, which we use as a proxy for gray matter density. All MRI volumes were then resampled to 2 mm^3^ resolution and smoothed using a FWHM 5 mm kernel implemented in SPM8.

To identify regions of reduced gray matter density in bvFTD we performed a two-samples *t*-test in SPM8 for bvFTD patients relative to healthy seniors. For this analysis we used a FDR-corrected height threshold (*q* < 0.05) and only accepted clusters that survived a 100 adjacent voxel extent. To relate gray matter density to behavioral performance we performed a two-stage analysis. First, we defined regions of interest (ROI) that consisted of voxels that had significantly reduced gray matter density relative to healthy seniors and then identified the conjunction of reduced gray matter density in bvFTD patients and the regions of activation observed in Experiment 1. We then exported the mean gray matter density within each overlapping ROI. Second, we performed bivariate correlations relating response times for ambiguous sentences to the gray matter density within each ROI (*p* < 0.05 Bonferroni corrected for multiple comparisons). We constrained our correlation analyses to the conjunction of disease and fMRI ROIs in order to limit our interpretations to areas of known disease. It would otherwise be difficult to interpret a significant correlation outside these overlapping regions since variance in gray matter density may be related to disease-independent factors such as aging or individual differences in neuroanatomical structure.

### Results

#### Behavioral results

**Figure 4:**
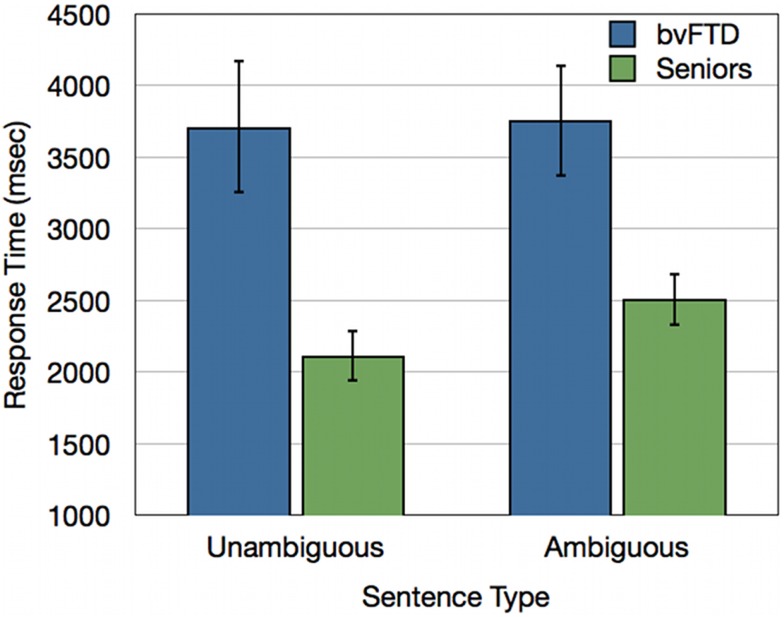
**Response times (ms) for bvFTD patients and healthy seniors for unambiguous baseline sentences and ambiguous doubly-quantified sentences**.

We performed an ANOVA of response times with a Sentence Type × Group design. This revealed a significant Sentence Type × Group interaction [*F*(1, 30) = 4.58; *p* < 0.05]. bvFTD patients did not show a difference in response times across ambiguous and unambiguous sentences, while healthy seniors showed significant processing cost of an average of 393 ms for ambiguous sentences relative to unambiguous sentences. A main effect also revealed that bvFTD patients were overall slower to respond to sentence materials than healthy seniors [*F*(1, 30) = 11.19; *p* < 0.005] (see Figure 4).

#### Volumetric MRI results

**Figure 5:**
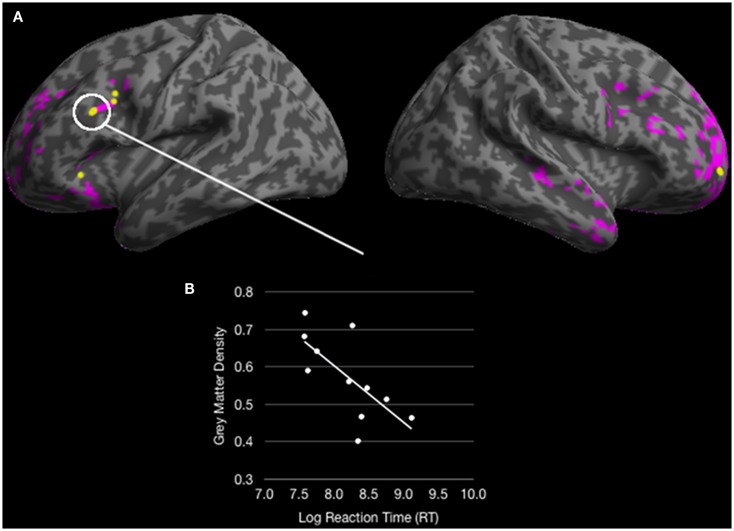
**(A)** Colored regions (pink and yellow) represent regions of significantly reduced gray matter density for behavioral variant frontotemporal dementia (bvFTD) patients relative to healthy seniors (*p* < 0.05 FDR; 100 voxel extent) and yellow regions represent overlapping reduced density with fMRI activation for healthy young adults in Experiment 1; **(B)** A significant correlation between an overlapping region in dorsolateral prefrontal cortex correlates with response times for ambiguous doubly-quantified sentences.

An analysis of volumetric MRI revealed significantly reduced gray matter density for bvFTD patients relative to healthy seniors in frontal and temporal cortex, including: bilateral rPFC, rostromedial PFC, right IFC, left dlPFC, left insula, left anterior cingulate, left medial temporal cortex, bilateral anterior temporal cortex, and right plTC (see purple and yellow regions in Figure 5A and Table 3). Among these regions of reduced gray matter density in bvFTD, we observed five ROIs that overlapped with regions of fMRI activation during ambiguous doubly-quantified sentence interpretation reported in Experiment 1. These regions, illustrated in yellow in Figure 5A (see also Table 4), include right rPFC, two clusters in left dlPFC, left IFC, and left ventrolateral prefrontal cortex.

**Table 3:** **Regions of significantly reduced gray matter density in bvFTD patients relative to demographically matched healthy seniors (*p* < 0.05 FDR-corrected; 100 voxel extent)**.

Neuroanatomic Region (BA)	L/R	Peak MNI coordinate			*Z*-score	Voxels
		*x*	*y*	*z*	
Rostromedial prefrontal (10)	M	−6	50	16	4.63	4163
Rostral prefrontal (10)	L	−26	52	4	4.31	297
Rostral prefrontal (10)	L	−26	44	30	3.96	254
Inferior frontal (44/45)	R	50	4	22	3.81	385
Dorsolateral prefrontal (9)	L	−40	2	38	4.11	154
Anterior cingulate (24)	L	−18	−10	24	3.77	111
Insula (12)	L	−38	12	4	3.29	135
Medial temporal (21)	L	−38	−24	−16	4.17	578
Anterior temporal (38)	L	−36	8	−26	3.86	176
Anterior temporal (38)	R	42	10	−30	3.76	347
Posterior-lateral temporal (22)	R	52	−26	0	3.54	110

**Table 4:** **Regions of overlap between fMRI activation in healthy young adults and reduced gray matter density in bvFTD patients relative to healthy seniors**.

Neuroanatomic Region (BA)	L/R	Centroid MNI Coordinate		
		x	y	z
Dorsolateral prefrontal (9)	L	−46	6	38
Rostral prefrontal (10)	R	22	58	−6
Inferior frontal (45)	L	−36	18	22
Ventrolateral prefrontal (47)	L	−34	30	−2
Dorsolateral prefrontal (9)	L	−42	20	30

We performed correlation analyses to relate bvFTD patients’ response times to ambiguous sentences with reduced gray matter density in each ROI overlapping with the fMRI results from Experiment 1. These analyses revealed that response times to ambiguous sentences were related to gray matter density in left dlPFC [*r* = −0.68; *p* < 0.05 Bonferroni corrected] (see Figure 5B). Correlations of ambiguous sentence response times in the remaining ROIs did not approach significance (*p* > 0.1 Bonferroni corrected). However, an analysis evaluating gray matter density in the left dlPFC revealed that density in this ROI was related to the other ROIs (all *p* < 0.05 Bonferroni corrected) suggesting that an overall degradation of a network involved in the interpretation of doubly-quantified sentences may contribute to bvFTD patients’ compromised sensitivity to ambiguity.

### Experiment 2 interim discussion

Healthy seniors, like young adults in Experiment 1, responded slower to ambiguous than unambiguous sentences and these results are consistent with the hypothesis that additional processing costs are incurred to support the comprehension of doubly-quantified sentences. However, non-aphasic patients with bvFTD did not demonstrate a processing cost associated with ambiguity. We found that bvFTD patients’ response times to ambiguous doubly-quantified sentences were related to disease in left dlPFC, a region recruited by young adults to support ambiguous sentence processing demands. This dlPFC region was also correlated with the magnitude of atrophy in additional frontal regions and suggests that bvFTD patients may have a degraded network extending beyond peri-Sylvian cortex that contributes to their insensitivity to ambiguity associated with doubly-quantified sentences.

## General Discussion

Many utterances in our day-to-day speech are ambiguous. Nevertheless, we are able to resolve these ambiguities and communicate effectively. In the present study, we found that this depends only in part on the core left peri-Sylvian sentence processing network. We obtained converging evidence from an fMRI study of healthy adults and a bvFTD patient study that extra-Sylvian regions additionally contribute to the processing resources required to resolve ambiguous doubly-quantified sentences.

In the fMRI study we observed recruitment of four cortical regions that extend beyond the traditional peri-Sylvian language network, including right rPFC, dorsomedial prefrontal cortex, a cluster extending into left dlPFC, and left parietal cortex. Together, these regions have often been implicated as contributing to a multi-demand network that supports the upregulation of resources to support a variety of cognitive tasks (Duncan, 2010). BOLD resting studies have also suggested that dlPFC and parietal cortex activation together contribute to an executive-control network that correlates with executive task performance (Seeley et al., 2007). We observed that left dlPFC atrophy was related to bvFTD patients’ response times to ambiguous doubly-quantified sentences and that the magnitude of left dlPFC atrophy was correlated with atrophy in the other frontal regions that overlapped with fMRI task activation. We interpret these findings as evidence that extra-Sylvian regions contribute to the processing resources required to interpret ambiguous doubly-quantified sentences and that atrophy in these regions result in an insensitivity to the ambiguous nature of these sentences. While the current study cannot resolve the specific mechanistic nature that each of these regions contributes to ambiguity resolution we provide a potential account for each region in the context of previous studies.

We observed activation of dlPFC during the interpretation of ambiguous sentences in the fMRI study of healthy adults, and this overlapped with a dlPFC area of gray matter atrophy in bvFTD. Moreover, we found a correlation between bvFTD patient performance and gray matter atrophy in dlPFC. Previous work has associated this area with the interpretation of sentences containing a temporary structural ambiguity (Novais-Santos et al., 2007). dlPFC is within the spectrum of prefrontal brain regions involved in decision-making and ambiguity resolution (Novais-Santos et al., 2007; Hoenig and Scheef, 2009; McMillan et al., 2011, 2012a). More recently, non-linguistic studies have suggested that dlPFC may be involved in probabilistic estimation (Casey et al., 2001; Scheibe et al., 2010), and in a pronoun resolution task dlPFC activation was modulated by the likelihood of a pronoun referring to a referent (McMillan et al., 2012a). In the current study, dlPFC activation may also be related to probability since “all” has a preferred, or probabilistically more likely, collective interpretation, but future work is required to investigate the specific role of this region in ambiguity resolution.

An alternate hypothesis is that dlPFC activation is related to a difficult working memory challenge. Some fMRI studies have suggested that the magnitude of dlPFC activation supports increased working memory demands. For example, when the *N*-back task requires participants to maintain one or two items in working memory activation is typically observed in more inferior frontal regions, but when participants need to maintain three or more items activation extends dorsally to include dlPFC (Braver et al., 1997; Botvinick et al., 2001). A counter-argument for this pattern of activation is that dlFPC instead supports a strategic manipulation rather than increased working memory capacity (Smith et al., 2001). bvFTD patients have working memory deficits (Libon et al., 2007) and this can potentially interfere with their comprehension of sentences with lengthy material (Cooke et al., 2003). While we did not explicitly manipulate working memory demands in these stimuli, the experimental stimuli were matched with the baseline stimuli for sentence length and only differed by a single word. Another possible explanation of dlPFC activation is that, together with anterior cingulate activation, these regions may be contributing to response selection during task performance rather than the properties of the stimulus sentences (Cohen et al., 2000; Botvinick et al., 2001, 2004).

In the fMRI study, we also found that rPFC is recruited during the interpretation of doubly-quantified sentences. rPFC has been associated with strategic planning and sub-goal decision-making in non-linguistic contexts that depend on several contingencies (Koechlin et al., 2003; Badre and Wagner, 2007; Koechlin and Hyafil, 2007; Badre et al., 2009). This area has been associated with sub-goal and episodic goal monitoring, relational integration, and shifting from internal to external attention. We forward the hypothesis that rPFC is recruited to perform a similar role in the interpretation of doubly-quantified sentences. In the present study, comprehension depends in part on appreciating that a sentence can have multiple meanings, that sub-goal planning may play a role in interpreting the contingency relationship between the interpretive bias associated with a quantifier and the size of the object in the sentence, and that “all” is biased toward a collective interpretation that maps on to a larger object. We hypothesize that this complex process may be coordinated in part by rPFC.

Additional evidence consistent with the hypothesis that rPFC is involved in decision-making during the processing of ambiguous sentences, even though it is not part of the core left peri-Sylvian language network, comes from the study of patients with bvFTD. These patients are not aphasic: their speech is generally well-structured grammatically, and they have minimal difficulty interpreting word meaning, although they do have some difficulty appreciating the hierarchical structure of lengthier narratives (Ash et al., 2006; Farag et al., 2010) and they are impaired at lexical selection when trying to establish common ground with a conversational partner (McMillan et al., 2012b). Despite the absence of aphasia, these patients appear to have difficulty appreciating the ambiguity present in a doubly-quantified sentence. In contrast we observed that healthy young adults (Experiment 1) and healthy seniors (Experiment 2) exhibit a processing cost when interpreting doubly-quantified sentences.

We also observed activation of inferior parietal cortex. One possibility relates this to the quantifier component of a double-quantified sentence. For example, previous investigations of quantifier comprehension demonstrated inferior parietal cortex activation, supporting a numerosity-related mechanism for understanding quantifiers (McMillan et al., 2005, 2006, 2011; Morgan et al., 2011; Heim et al., 2012). However, parietal activation in previous work was associated with first-order and higher-order quantifiers more than Aristotelean quantifiers like “all” (Troiani et al., 2009a). A related possibility is that the magnitude of the objects (e.g., the size of a puddle versus a lake) contributed to the comprehension of these sentences, and comprehension of the magnitude dimension of these objects may have in part played a role in parietal activation (Heim et al., 2012). This is less likely since magnitude information was present in both the experimental and the baseline stimuli, although detailed appreciation of magnitude may have been needed only for the ambiguous stimuli. An alternate possibility is that parietal activation may be related in part to a component of the decision-making mechanism that integrates several elements of a decision to find an optimal solution (Platt and Glimcher, 1999; Huettel et al., 2005; McMillan et al., 2012a). Additional work is needed to establish the basis for parietal activation in this study.

The BOLD fMRI study additionally demonstrated activation of peri-Sylvian brain regions during the processing of doubly-quantified sentences, including both left plTC and left IFC. These regions are commonly implicated in supporting a core language network (Friederici et al., 2003; Grodzinsky and Friederici, 2006; Grodzinsky and Santi, 2008). It is possible that this core language network is upregulated in part to support the working memory resources needed to disambiguate ambiguous sentences (Mason et al., 2003), but importantly our findings suggest that additional processing resources are also required to support the processing costs associated with interpreting doubly-quantified sentences. Critically, bvFTD patients’ limitations were not related to the magnitude of disease in these core language regions.

We emphasize that our findings are based on converging data from multiple sources, including both fMRI and patient data. This is important because fMRI studies show correlated activation of brain regions contributing to task performance in healthy adults, but do not identify the subset of these regions that are necessary for performance – in this case, the brain regions that contribute to disambiguating a doubly-quantified sentence. Even with a high-level baseline condition such as we used in the present study, there may be other factors contributing to the difference between ambiguous and unambiguous sentences that are not accounted for in the “cognitive subtraction” logic underlying the fMRI study. Perhaps even more crucial is evidence from patients with disease in brain regions critical to testing our hypothesis about the disambiguation of doubly-quantified sentences. This is critical because these patients have difficulty interpreting a sentence even though they do not have aphasia. This emphasizes the apparent contribution of resources that are not part of the core sentence processing network to the interpretation of ambiguous sentences.

There are several shortcomings in our study, and these should be kept in mind when interpreting the results. First, we did not study the role of the parietal lobe in non-aphasic patients with parietal disease to see if they too have difficulty interpreting ambiguous sentences. This would have helped establish the basis for parietal activation, and determined whether parietal activation seen in the fMRI study is related to decision-making or magnitude. Second, it will be important to confirm our findings with another kind of ambiguous sentence to evaluate the specific mechanistic role of the implicated extra-Sylvian neuroanatomic regions. With these caveats in mind, our findings are consistent with the claim that frontal and parietal brain regions outside of the traditional peri-Sylvian sentence processing network contribute to the disambiguation of doubly-quantified sentences.

## Conflict of Interest Statement

The authors declare that the research was conducted in the absence of any commercial or financial relationships that could be construed as a potential conflict of interest.

## Footnotes

^1^https://sourceforge.net/projects/neuropipedream/

^2^http://www.picsl.upenn.edu/ANTS/
